# Genetic mechanisms underlying the methylation level of anthocyanins in grape (*Vitis vinifera *L.)

**DOI:** 10.1186/1471-2229-11-179

**Published:** 2011-12-15

**Authors:** Alexandre Fournier-Level, Philippe Hugueney, Clotilde Verriès, Patrice This, Agnès Ageorges

**Affiliations:** 1 INRA, UMR 1097 Diversité et Adaptation des Plantes Cultivées, 34060 Montpellier, France; 2 INRA, UMR 1131 Santé de la Vigne et Qualité du Vin, F-68000 Colmar, France. Université de Strasbourg, UMR 1131, 67000 Strasbourg, France; 3 INRA, UMR 1083 Sciences pour l'OEnologie, 34060 Montpellier, France; 4 Department of Ecology and Evolutionary Biology, Brown University, Providence, RI 02912, USA

## Abstract

**Background:**

Plant color variation is due not only to the global pigment concentration but also to the proportion of different types of pigment. Variation in the color spectrum may arise from secondary modifications, such as hydroxylation and methylation, affecting the chromatic properties of pigments. In grapes (*Vitis vinifera *L.), the level of methylation modifies the stability and reactivity of anthocyanin, which directly influence the color of the berry. Anthocyanin methylation, as a complex trait, is controlled by multiple molecular factors likely to involve multiple regulatory steps.

**Results:**

In a Syrah × Grenache progeny, two QTLs were detected for variation in level of anthocyanin methylation. The first one, explaining up to 27% of variance, colocalized with a cluster of Myb-type transcription factor genes. The second one, explaining up to 20% of variance, colocalized with a cluster of *O-*methyltransferase coding genes (AOMT). In a collection of 32 unrelated cultivars, *MybA *and *AOMT *expression profiles correlated with the level of methylated anthocyanin. In addition, the newly characterized *AOMT2 *gene presented two SNPs associated with methylation level. These mutations, probably leading to a structural change of the AOMT2 protein significantly affected the enzyme specific catalytic efficiency for the 3'-*O-*methylation of delphinidin 3-glucoside.

**Conclusion:**

We demonstrated that variation in methylated anthocyanin accumulation is susceptible to involve both transcriptional regulation and structural variation. We report here the identification of novel AOMT variants likely to cause methylated anthocyanin variation. The integration of QTL mapping and molecular approaches enabled a better understanding of how variation in gene expression and catalytic efficiency of the resulting enzyme may influence the grape anthocyanin profile.

## Background

Anthocyanins represent a major group of the flavonoid family, consisting of small water-soluble molecules stored in vacuoles. As widespread plant secondary metabolites, they are responsible for red and blue colors of many plant tissues [1]. Anthocyanins, as major pigments, play important roles in plant reproduction by attracting pollinators and seed dispersers and in protection from photo-oxidative stress [2]. In crops, ornamental plants, and fruits, they constitute a key trait for the aesthetic quality of the product. In grape, they play a crucial role as they participate in both wine color and organoleptic properties due to their complex interactions with other phenolic compounds, as well as with proteins and polysaccharides [3].

**Figure 1:**
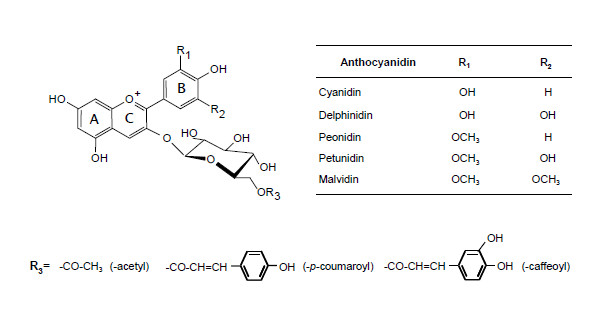
**Chemical structure of anthocyanins from grape berries (*Vitis vinifera L.)***.

The anthocyanins identified in *Vitis vinifera *are 3-*O-*monoglucosides (3-glc) and 3-*O-*acyl monoglucosides derived from the five main anthocyanidins--delphinidin (Dp), cyanidin (Cy), peonidin (Pn), petunidin (Pt) and malvidin (Mv)--, which differ from each other in the number and position of the hydroxyl and methoxyl groups located on their B-ring (Figure 1): delphinidin and cyanidin being unmethylated, peonidin and petunidin mono-methylated and malvidin di-methylated. Methylation by S-adenosyl-L-methionine (SAM) dependent-*O-*methyltransferase plays an important role in modifying the structure of plant secondary metabolites. For anthocyanins, methylation stabilizes the phenolic B ring, thus reducing the reactivity of the overall molecule [4], and increases water solubility, thus reinforcing its color properties [5,6]. The color of anthocyanins greatly depends on the number of hydroxyl groups: the larger the number of groups, the bluer the color. On the other hand, the *O-*methylation of anthocyanins has a reddening effect.

The anthocyanin biosynthesis pathway has been extensively characterized in numerous plant species [7,8]. In grapevine, the core structural genes of the anthocyanin pathway leading to the synthesis of Dp 3-glc and Cy 3-glc have been cloned and characterized [9,10]. Recently, two anthocyanin *O-*methyltransferases (AOMT and FAOMT) able to methylate anthocyanins *in vitro *and *in vivo *have been identified [11,12]. Anthocyanin biosynthesis is controlled by Myb-bHLH transcriptional complexes that activate the expression of structural genes involved in the late steps of the pathway [7,13-16]. In particular, the *VvMybA *genes induce the transcription of *UFGT *and *AOMT *in colored tissues [17-19] and were shown to control anthocyanin accumulation in grape [20,21]. While anthocyanin methylation in grapes is altered by environmental and cultural conditions [22,23], it is also strongly affected by genetic factors. Indeed, both the total amount of anthocyanins and the relative abundance of single anthocyanins are extremely variable among red--to blue-skinned cultivars. In spite of this variability, mono--and di-methyl derivatives are largely predominant [24,25], malvidin 3-glucoside being the main anthocyanin in most cultivars and peonidin 3-glucoside usually being less abundant [26,27]. Genes encoding flavonoid 3'--and 3'5'--hydroxylases involved in anthocyanins hydroxylation [28,29] or genes encoding *O-*methyltransferase involved in anthocyanins methylation [11,12,30] can potentially generate differences in anthocyanin composition. The availability of the full genome sequence [31,32] as well as broad genetic resources will help us to further clarify the mechanisms involved in anthocyanin biosynthesis in grape.

The objective of this study was to understand the architecture of the genetic control of anthocyanin methylation in grape and to improve our understanding of the complex factors involved in the regulation of anthocyanin methylation. Two QTLs for anthocyanin methylation variation were identified: one colocalized with a *MybA *gene cluster and the second colocalized with a cluster of three putative *AOMT *genes. This work aims to validate the role of the latter in anthocyanin methylation and to identify the molecular factors underlying genetic variation. The combination of forward genetic approaches (pedigree and population-based) with functional genomics and enzymology allowed the description of the complex regulation of an anthocyanin-*O-*methyltansferase gene cluster as a major player for the control of anthocyanin methylation in grape.

## Results

### Variation in methylated anthocyanin content

**Figure 2:**
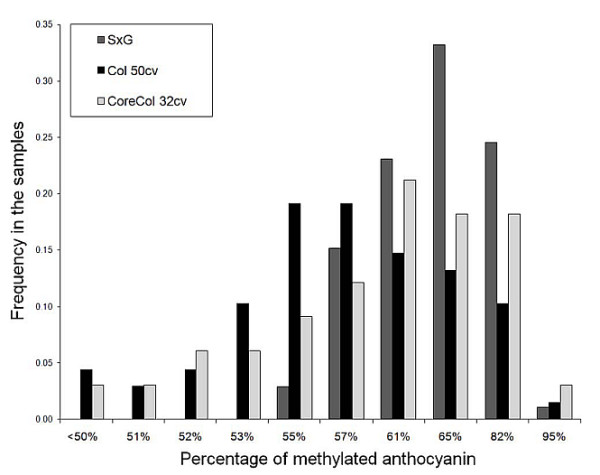
**Distribution of the *MT *variable in the samples used in QTL and association mapping**. The *MT *variable is expressed as the logarithm of the ratio of methylated/non-methylated anthocyanins in mg of anthocyanin per g of fresh berry skin. SxG: 191 individuals from the Syrah × Grenache progeny used in QTL mapping. Coll 50 cv: random sampling of 50 colorful grape cultivars from the original agromorphological core-collection (Barnaud et al. [33]). Core-Coll 32 cv: core-collection designed from the Coll 50 cv to optimize the capture of *MT *variability.

In order to investigate the genetic bases of anthocyanin methylation, three plant collections were analyzed: (1) a Syrah × Grenache (SxG) progeny of 191 genotypes replicated in two blocks, (2) a collection of 50 colored genotypes from the Vassal germplasm collection (Coll 50 cv, INRA, Domaine de Vassal, France), and (3) a subset of 32 genotypes selected to optimally represent the diversity in anthocyanin methylation level of the original 50 individuals sample with a smaller number of individuals (Core-Coll 32 cv). Anthocyanin composition differed dramatically both in the SxG progeny (Additional file 1) and in the collection of natural diversity (data presented for Core-Coll 32 cv, Additional file 2). After a *log*-transformation, distribution of methylated anthocyanin (MT) was normal in the three samples (*P*-val > 0.05). The SxG progeny showed extensive variation in levels of MT, ranging from 50% to 97% of methylated anthocyanins with a MT variance of *σ*^2^_MT SxG _= 0.42 (Figure 2). The collection of 50 colored grape varieties (Coll 50 cv) showed variation ranging from 31% to 97% of methylated anthocyanins with a MT variance of *σ*^2^_MT 50 cv _= 0.58. The variance was increased to *σ*^2^_MT 32 cv _= 0.89 in the Core-Coll 32 cv.

### QTL mapping of anthocyanin methylation level

**Figure 3:**
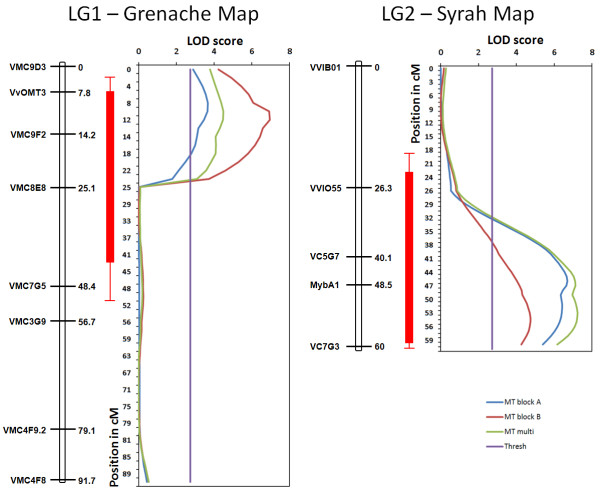
**Position and linkage intensity of the QTL associated with the MT trait**. The two QTLs were detected on the SXG mapping population on the Grenache parental map and on the Syrah parental map, respectively. Confidence intervals correspond to the 90% and 99% probability of presence of the QTLs after 1000 bootstrap resamplings. The QTLs presented were found for the A and B blocks and using multi-trait detection.

QTL detection was performed either on the two field blocks taken separately or combined using a multi-trait procedure. In both cases, this allowed the consistent identification of two QTLs for the anthocyanin methylation level. The first QTL, explaining 27% of the phenotypic variance for anthocyanin methylation, was located on Linkage Group 2 (LG2) between the markers VMC5G7 and VMC8C2 (Figure 3). This QTL was detected on the Syrah parental map and supported with a maximum LOD of 7.49 for the multi-trait detection. It was also detected on both blocks taken separately. This QTL, which colocalized with a QTL previously identified as controlling the content of anthocyanins in grape berry [20], corresponded to the *VvMybA *genes cluster, thus controlling together anthocyanin methylation and total accumulation. The second QTL was identified on LG1 of the Grenache parental map between markers VMC9E3 and VMC8E8, explaining up to 20% of the phenotypic variance with a maximum LOD of 6.96 (Figure 3). In the present study, we focused on this second QTL, which was specific to anthocyanin methylation. The marker pOMT2, the closest to the LG1 QTL, showed a difference of 11.8% between homozygous combination of alleles leading to high level of MT and heterozygous combination at pOMT2 leading to low level of MT (Additional file 3).

### Candidate genes selection in the QTL on LG1

**Figure 4:**
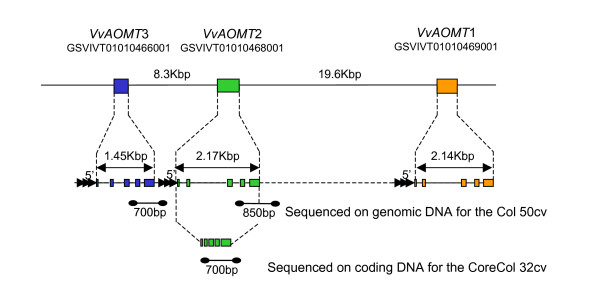
**Genomic structure of the open reading frames of the *VvAOMT *genes cluster on chromosome 1**. The continuous lines represent sections drawn at the right scale; dashed lines represent change in scale. Lines between dots represent the sequenced fragments. The filled boxes indicate the exons. Data correspond to the 12X genome annotation available from the Genoscope Grape Genome Browser database http://www.genoscope.cns.fr/externe/GenomeBrowser/Vitis/.

The physical interval of 1 Mbp surrounding the maximum LOD of the QTL of LG1 encompassed 46 predicted unigenes with an identity score superior to 5 (Additional file 4). Among this unigenes set, a cluster of three genes encoding putative anthocyanin *O-*methyltransferases (AOMT) within a 33 Kbp region was identified, referred to hereafter as *VvAOMT1, VvAOMT*2 and *VvAOMT*3 (Figure 4). *VvAOMT1 *encoded a protein that is identical to the AOMT from Syrah shown to methylate anthocyanin both *in vitro *and *in planta *[11] and similar to the FAOMT from Cabernet Sauvignon shown to methylate anthocyanin *in vitro *[12]; therefore, we narrowed the study to these three candidate genes. The three genes have similar structures, with five small exons ranging from 51 bp to 294 bp and four introns (Figure 4). The deduced amino acid sequences of the three OMTs showed high sequence similarity (Additional file 5) with a minimum pairwise identity score of 90% at the genomic level and of 96% at the transcript level. In addition, there was no other protein showing more than 20 amino acids with 80% similarity to AOMT in the grape genome, showing evidence for *VvAOMT *being a single family of paralogs. The corresponding transcripts were 708 bp long and very similar to *VvAOMT1*. All three genes were thus potentially functional. The 3' non-coding ends of *VvAOMT *genes appeared less conserved than the coding region, and the only robust distinction between the three isogenes thus relied on divergent non-coding 3' ends. Isogene-specific primers could be designed for *VvAOMT*2 and *VvAOMT*3 cDNA, but not for *VvAOMT*1 cDNA, due to sequence homology with the two other isogenes and a shorter 3' end. Using specific primers for *VvAOMT*3, no expression could be detected in mature berries of Syrah, Joubertin and Petit Bouschet, suggesting that this isogene was not expressed in mature red berries (Additional file 6). Although this does not demonstrate the non-functionality of *VvAOMT*3, it justified the further consideration of *VvAOMT1 *and *VvAOMT*2, as candidate genes in association genetic analysis both expressed in mature berries.

### Effect of the *VvAOMT *and *VvMybA *expression on methylated anthocyanins

To assess the relative influence of *VvAOMT *and *VvMybA *genes, which potentially underlie the QTLs on LG1 and LG2, respectively, we measured their respective transcript accumulation in the Core-Coll 32 cv, maximizing the diversity for methylated anthocyanins. We analyzed the level of expression of *VvAOMT *(*eAOMT*), which combines the expression of the three isogenes *VvAOMT1, VvAOMT*2 and *VvAOMT*3 and of *VvMybA *(*eMybA*), which combines the expression of the two isogenes *VvMybA*1 and *VvMybA*2. Both *eAOMT *and *eMybA *appeared to follow continuous variation among the 32 cultivars, showing no evidence of regulation by a single major polymorphism. *eAOMT *appeared to be highly correlated with the methylated anthocyanin content of the berry skin (*ρ *= 0.58, *P*-val < 0.001; Table 1). *eMybA *appeared correlated with total anthocyanin content, but not with the methylated anthocyanin content (Table 1). Finally, the methylated anthocyanin content was better predicted when the effects of *eAOMT *and *eMybA *were considered jointly in a multivariate regression model (Table 2), suggesting that the expression level of both genes had an effect on anthocyanin methylation, probably with a direct influence of *eAOMT *and an indirect influence of *eMybA*.

**Table 1:** Correlation between the anthocyanin content and *VvMybA, VvAOMT *and specific *VvAOMT2 *expression

	Total Antho	*eMybA*	MT	e*AOMT*	e*AOMT2*
Total Antho	1				
e*MybA*	0.48	1			
	0.042*				
MT	-0.01	-0.17	1		
	*0.9636*	*0.35*			
e*AOMT*	0.31	0.27	0.58	1	
	*0.0812*	*0.1348*	0.0005**		
e*AOMT2*	0.04	-0.04	0.02	0.40	1
	*0.8356*	*0.8182*	*0.93*	0.0229*	

Total Antho: total anthocyanin content, MT: level of methylated anthocyanin, *eMybA*: global *VvMybA *expression level, *eAOMT*: global *VvAOMT *expression and *eAOMT2: *specific *VvAOMT2 *expression in the Core-Coll 32 cv sample. The Pearson's ρ values are presented in plain text while their associated *P*-values are shown in italic (* < 5% and ** < 1%)

**Table 2:** Effects of *VvAOMT *and *VvMybA *global expression on the *MT *variable in the Core-Coll 32 cv

N of variables	ajusted R^2^	BIC	Variables in the model	
1	0.31	-23.17	*eOMT*	
2	0.41	-26.72	*eOMT*	*eMybA*
			*0.31*	*0.1*

Both variables (*eAOMT *and *eMybA*, respectively) show independent significant effect in a multiple regression test, the number *in italic *corresponds to the partial R-squared of each effect tested

### Association mapping with *VvAOMT1 *and *VvAOMT2*

To isolate the particular effect of the two *VvAOMT *candidate genes, we partially sequenced both genes in the collection of 50 colored varieties (genomic DNA fragments corresponding to 700 bp of 3'end of *VvAOMT1 *and 850 bp of 3'end of *VvAOMT*2; Figure 4). This allowed detecting polymorphisms on genomic DNA in the two last exons and in the 3'UTR specifically for both *VvAOMT *candidates (high sequence homology and shorter 3'UTR for *VvAOMT1 *did not allow specific amplification at the complete coding sequences). On *VvAOMT1*, we identified 15 SNPs and 2 InDels, and, on *VvAOMT2*, we identified 19 SNPs and 1 InDel, giving rise to 9 non-synonymous mutations. We tested the association of these polymorphisms with the level of methylated anthocyanins using the EMMA software [34]. Six SNPs were identified as associated with variation in anthocyanin methylation in *VvAOMT*2 and none in *VvAOMT1*. The significantly associated polymorphisms with *P*-value < 0.05 and the test statistics are presented in Table 3. SNP Y522 did not change the amino acid sequence of the gene, and the five other associated polymorphisms were located in the 3'UTR part of the *VvAOMT*2 gene, thus not affecting the protein structure. All six associated SNPs showed significant LD, with one LD block, including SNPs Y522, Y717, Y741 and W774 (pairwise r ^2 ^= 0.5-1), and another LD block, including M843 and InDel909 (pairwise r ^2^= 0.6). However, given the high level of polymorphism at both loci, we expected that if there was functional variation elsewhere in the *VvAOMT1 *sequence, we would have genotyped at least one SNP significantly associated through LD with the causal variation. In addition to the two blocks of LD reported for *VvAOMT2*, two blocks of high LD were identified on *VvAOMT1 *(Additional file 7). However, no intergenic LD was significant between *VvAOMT1 *and *VvAOMT2*, showing a rapid decay of LD in the genomic fragment including these genes. As *VvAOMT2 *was the only isogene showing polymorphism association with anthocyanin methylation level, we only retained *VvAOMT2 *as candidate gene.

**Table 3:** Association genetics tests performed on *VvAOMT*2 polymorphisms for the level of methylated anthocyanin

Samples	Marker	df Marker	F test	*P-value*	R^2 ^Model	R^2 ^Marker
Coll 50 cv	Y522	1	12.51	0.0041	0.61	0.41
	Y717	1	5.67	0.0347	0.46	0.26
	Y741	1	10.21	0.0085	0.51	0.46
	W774	1	5.67	0.0347	0.46	0.26
	M843	2	5.75	0.0195	0.61	0.41
	InDel909	2	4.22	0.0435	0.55	0.35
Core-Coll	Y127	2	7.80	0.0039	0.53	0.43
32 cv	W388	2	7.80	0.0039	0.53	0.43
	Y741	2	7.80	0.0039	0.53	0.43

Tests were performed using genomic DNA sequence fragments in the Coll 50 cv and coding DNA sequence in the Core-Coll 32 cv. Only the significant tests are shown. Df: degree of freedom, R^2^: percentage of explained variance

### Specific expression of *VvAOMT2*

Isogene-specific primers were designed to measure the specific expression of the candidate gene *VvAOMT*2 in *(1) MybA1 *transformed grapevine hairy roots accumulating anthocyanin [18] and in *(2) *the Core-Coll 32 cv (Additional file 2). As we did not succeed in designing specific primers for *AOMT1*, we could only compare the global expression e*AOMT *(*VvAOMT1 *plus *VvAOMT*2) using non specific primers with the specific expression *VvAOMT*2. In hairy roots over-expressing *MybA1*, the expression of *VvAOMT*2 showed a 5.5-fold increase compared to the control, while the level of *eAOMT*, showed a 75-fold increase (Additional file 8). Although the effect of *VvMybA*1 on *VvAOMT*2 cannot be neglected, these results suggest that the effect of *VvMybA*1 on *VvAOMT*2 is limited. In the 32 cultivars subcore-collection, no significant correlation was found between the expression level of *VvAOMT*2 and the ratio of methylated anthocyanins. Moreover, no significant effect of *eMybA *was observed on the expression of *VvAOMT*2 (Table 1). However, even if the specific expression level of *VvAOMT*2 is correlated with global *eAOMT *(*ρ *= 0.40, *P*-val = 0.023) and has a significant regulatory effect of *eAOMT *on the level of methylation, in the absence of correlation between *VvAOMT*2 expression and MT, we concluded that *VvAOMT*2 expression had no direct regulatory effect on anthocyanin methylation.

### Sequence analysis of the *VvAOMT2 *gene

We fully sequenced the *VvAOMT*2 cDNA of the Core-Coll 32 cv and identified 30 polymorphisms in the ORF, including 26 SNPs and 4 InDels (Additional file 9). As the genomic and cDNA fragments sequenced for *VvAOMT*2 partially overlapped, 8 were common to those already identified in the 50 cultivars sample, including the 4 most associated SNPs presented in Table 3. This new polymorphism set was tested again for association with methylated anthocyanin level in the Core-Coll 32 cv and only three associations were found to be significant: Y741 was again strongly associated, as well as two new SNPs, Y127 and W388. These three SNPs showed complete linkage disequilibrium (r ^2 ^= 1) and their effects were thus confounded. In addition, the *VvOMT2 *sequence for the parents of the SxG population showed to be homozygous for the strong allele of W388 and Y741 in the Syrah parent and heterozygous in the Grenache parent, following the same segregation as the pOMT2 marker. This supported that the Grenache QTL on LG1 could be attributed to the segregation of VvOMT2 alleles in the mapping population.

Finally, in the best model including only expression profiles, *eAOMT *explained 31% of the variance while the single SNP Y127 or W388 in the association model explained 43% of variance for the same sample (Table 2 and Table 3). This strong effect was associated with a highly significant difference in anthocyanin methylation between the groups carrying the strong or the weak allele at SNPs Y127 and W388 (MT being on average 94.7% and 76.5% respectively). The SNPs Y127 and W388 corresponded to non-synonymous changes affecting the primary structure of the AOMT protein. Y127 led to a substitution from H to Y in position 43 of the amino acid chain and W388 led to a substitution from S to T in position 130 of the amino acid chain. Y127 appeared to generate a mutation within the second α-helix of the predicted protein secondary structure and W388, within the sixth predicted α-helix.

### Characterization of the allelic variants of *VvAOMT2*

**Figure 5:**
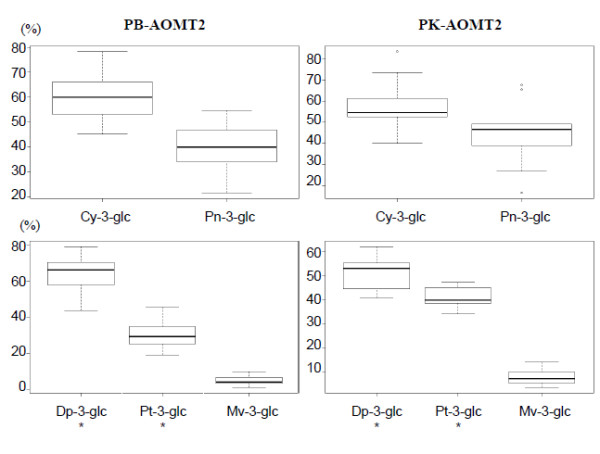
**Difference in catalytic efficiency between the PB-AOMT2 and PK-AOMT2 allelic variants**. Specific catalytic efficiency in performing 3'-and 3'5'-*O-*methylation was measured on either cyanidin 3-*O*-glucoside (Cy 3-glc) and delphinidin 3-*O*-glucoside (Dp 3-glc). Box plots indicate 5, 25, 50, 75 and 95 percentile, respectively. * indicates significant ANOVA test (*P*-value < 0.05)

Two grape varieties were selected for the homozygous presence of contrasting SNPs Y127 and W388 at the *VvAOMT*2 locus, representing two allelic variants of the corresponding AOMT2 protein. 'Petit Bouschet × Aramon n°4' (PB) presented the weak alleles T at Y127 and A at W388 and 'Papadiko' (PK) presented the strong alleles C at Y127 and T at W388. No other difference was detected in the coding region. Enzymatic tests performed with PB-AOMT2 and PK-AOMT2 indicated that both enzymes catalyzed *in vitro *the 3' *O-*methylation of anthocyanins with a catechol B-ring (3',4' di-OH) and the 3' and 5' *O-*methylation for those showing a pyrogallol (3',4',5' tri-OH) B-ring. However detailed analysis of PB-AOMT2 and PK-AOMT2 activity revealed substrate-specific differences in catalytic efficiency (Table 4). Indeed, when Cy 3-glc was used as a substrate, both PB-AOMT2 and PK-AOMT2 appeared to catalyze equally well the methylation of Cy 3-glc into Pn 3-glc. By contrast, when Dp 3-glc was used as a substrate, PB-AOMT2 showed a significantly reduced efficiency compared to PK-AOMT2 in producing Pt 3-glc (mean difference in relative final Pt 3-glc proportion of 9.7%, *P*-val = 0.02, Figure 5). Finally, both enzymes were able to convert Dp 3-glc in Mv 3-glc with similar efficiency although in small proportion.

**Table 4:** Substrate specificity of the recombinant AOMT2 proteins from Papadiko (PK-AOMT2) and from Petit-Bouschet × Aramon n°4 (PB-AOMT2)

Enzyme	Substrate	Products	relative amounts (%)
PK-AOMT2	Dp 3-glc	Dp 3-glc	51.1 (± 6.9) *
		Pt 3-glc	40.8 (± 4.0) *
		Mv 3-glc	8.1 (± 3.3)
	Cy 3-glc	Cy 3-glc	58.4 (± 12.2)
		Pn 3-glc	44.9 (± 15.4)
PB-AOMT2	Dp 3-glc	Dp 3-glc	63.6 (± 11.1) *
		Pt 3-glc	31.2 (± 8.6) *
		Mv 3-glc	5.3 (± 2.6)
	Cy 3-glc	Cy 3-glc	60.6 (± 9.3)
		Pn 3-glc	39.4 (± 9.2)

Reaction were essayed *in vitro *and products were identified according to their UV visible absorption spectra and retention time. Quantifications were based on peak areas at 520 nm. The data presented are the mean of 10 experiments performed with 3 independent protein preparations (± standard deviation), * indicates significance in ANOVA test (*P*-values < 0.05). Dp, delphinidin; Cy, cyanidin; Pt, petunidin; Pn, peonidin; Mv, malvidin; glc, glucoside

## Discussion

### Combining molecular physiology and genetics to dissect complex traits in plants

Major steps forward in the study of plant anthocyanin synthesis were made through molecular physiology and forward genetics experiments [7]. However, QTL and association mapping techniques have also proven to be relevant tools in the dissection of complex physiological traits, in particular for anthocyanin metabolism [35]. Since their large scale application in maize, only a few attempts to clone and validate the polymorphisms of the genes underlying variation have been successful [36]. In this work, we propose different approaches to draw a global picture of the regulation of anthocyanin methylation in grape. As a starting point, we performed a rational sampling of highly diversified phenotypes present in the Vassal germplasm repository (INRA, Domaine de Vassal, France) by using the M-method [37,38]. This method ensures the optimal capture of phenotypic diversity suitable to detect contrasted genetic effects. The use of QTL mapping and the colocalization of a *VvAOMT *cluster on chromosome 1, provided independent evidence supporting that the *VvAOMT *genes may play a key role in determining the level of methylated anthocyanins in grape. The availability of broad genetic resources together with the use of grape genomic tools [31] helped refine our QTL detection through gene expression and association mapping studies, demonstrating the likely involvement of both *cis*-regulation and newly characterized allelic variation in shaping a complex phenotype.

### The pleiotropic influence of *VvMybA *genes

The QTL for MT level detected on LG2 (explaining 35% of the variance) co-localized with a *VvMybA *genes cluster, supporting that *VvMybA *genes have an important effect on anthocyanin methylation. The results found in previous work with the same experimental design [20] showed nonetheless that this region better explained the total anthocyanin content (64% of variance explained) than the level of methylated anthocyanins. This suggested that this QTL, cosegregating with *VvMybA *genes cluster, mostly had an effect on total anthocyanin accumulation and by consequence, an indirect effect on anthocyanin methylation. The *VvMybA *genes control the transcription of key genes of the late steps of the anthocyanin biosynthesis: *UFGT *and *AOMT *[18]. Our study showed that *VvMybA*1 has a significant effect on global *VvAOMT*s expression level. *VvUFGT *catalyzes the glycosyl transfer on C3 of the anthocyanidin aglycone [19,39]. *VvAOMT *catalyzes the methylation of Dp 3-glc into both Pt 3-glc and Mv 3-glc, and that of Cy 3-glc into Pn 3-glc [11]. This pleiotropic effect of the *VvMybA *locus explains the relationship between the amount and the nature of the synthesized anthocyanins. This effect could also explain why black grape berries always contain large amounts of malvidin, the most methylated form of anthocyanidins [24-26]. *UFGT *and *AOMT *genes showed similar expression profiles [18,40] and to date, polymorphisms in their sequence were never associated with variation in anthocyanin content [9,11,13]. Thus, these genes were considered so far to be purely *cis*-regulated and to have no regulatory control on the anthocyanin pathway.

### Determining the influence of *VvAOMT *genes independently of *VvMybA *genes

We intended to identify regulation factors specific to the anthocyanin methylation level and independent of the total anthocyanin content. The MT variable and the total anthocyanin content were statistically independent, so the cosegregation of the QTLs for the two traits and the correlation between the *VvMybA1 *expression and MT were thus strictly due to biological effects. Nonetheless, genes specifically inducing differences in anthocyanin composition should either be independent of the expression of *VvMybA *or show *trans*-regulation or structural variation associated with anthocyanin methylation. In the search for such specific factors of the anthocyanin methylation process, the key was to find elements of variation that were significantly independent of the global anthocyanin *VvMybA cis*-regulated channel. Recently, a similar example was shown in Ipomoea for a dihydroxyflavonol reductase (a gene also involved in the anthocyanin pathway) where duplicated genes escaped the control network for greater adaptation [41]. We might also suspect this to be the case of *VvAOMT*2 and anthocyanin methylation.

### The *VvAOMT *genes cluster, the fate of a duplicated gene family

**Figure 6:**
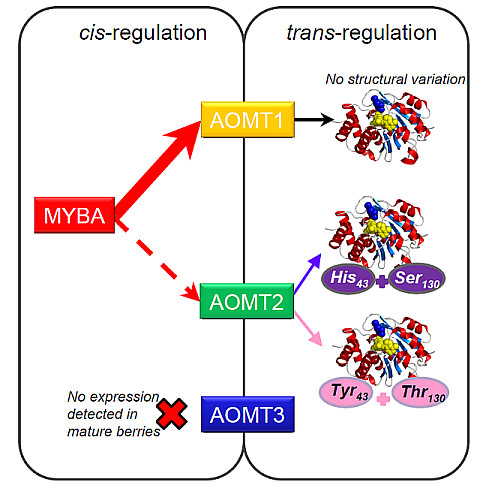
**Synthetic scheme of the regulation of anthocyanin methylation in grape berry**. The red arrows present the main influence of MybA protein *cis-*regulating AOMT1, the key enzyme involved in anthocyanin methylation. In addition, AOMT2, less influenced by MybA, *trans*-regulate anthocyanin methylation through allelic structural variation influencing enzymatic efficiency. Finally, *AOMT3 *expression was not detected in mature berries.

Grape varieties accumulating only non-methylated anthocyanins have never been described [24-26] and were also not identified in our study on a very diverse sample, necessarily involving the persistency of functional AOMT in the grape genome. Two mechanisms can generate such a pattern: a strong selective constraint on AOMT activity and a functional redundancy limiting the effect of deleterious mutations at *VvAOMT *locus. We identified 3 candidate genes encoding OMTs located on chromosome 1 in the confidence interval of the QTL, potentially all coding for functional proteins, which supports initial functional redundancy. In three genotypes with very contrasting MT phenotypes, only *VvAOMT1 *and *VvAOMT*2 were expressed in mature grape berries, no expression of *VvAOMT*3 being detected. The total expression of *VvAOMT *(*VvAOMT1 *and *VvAOMT*2 together) showed a high correlation with the level of methylation, while the specific expression of *VvAOMT*2 did not. We may thus hypothesize that *VvAOMT1 *is *cis*-regulated by the *VvMybA *genes, as reported in Ageorges et al. [17] and Cutanda-Perez et al. [18], and broadly determines the level of methylated anthocyanins. Conversely, *VvAOMT*2 expression was only marginally affected by *VvMybA *genes and showed structural variation, affecting anthocyanin methylation: the functional redundancy is decreasing as mutations accumulate. Although many other elements of variation may be involved in anthocyanin methylation, we may hypothesize a model as follows: *VvAOMT1 *is the fundamental isogene for anthocyanin methylation under strict functional constraint, while *VvAOMT*2 undergoing lesser functional constraint is responsible for fine and specific differences in the level of methylated anthocyanins (see proposed model Figure 6).

### The complex base of anthocyanin methylation in grape

Previous works in petunia and grape suggested that different OMTs were responsible for the *O-*methylation of anthocyanins at either 3' or 5' positions [42,43]. In contrast, Hugueney et al. [11] identified a bifunctional AOMT catalyzing 3' and 5' *O-*methylation, suggesting that AOMT alone would be sufficient to perform both methylation steps in grape berries. In this study, we showed that the two proteins encoded by allelic variants of *VvAOMT*2 were also able to perform *in vitro *3' or 3',5' methylation on Cy 3-glc and Dp 3-glc but in addition, we identified two SNPs associated with variation in the level of methylated anthocyanins. We showed that this polymorphism led to changes in substrate-specific catalytic efficiency, which only affects Dp 3-glc methylation into Pt 3-glc. Differential 3' *O-*methylation on trihydroxylated anthocyanin substrate due to protein variants may explain the quantitative differences observed *in planta*, after removing the effect of *VvAOMT1*. Furthermore, the comparison with the AOMT1 and FAOMT characterized previously [11,12] showed that AOMT2 variants characterized in this study tend to produce a lower proportion of Mv 3-glc, with a preferential catalysis of the 3' *O-*methylation. Indeed, both AOMT2 variants synthesized the same low relative proportion of Mv 3-glc (8.1% and 5.3%, respectively) while in similar conditions, AOMT1 was able to synthesize up to 34% of Mv 3-glc [11]. Finally, variation in catalytic properties together with potential differential expression is definitely capable of creating the complex pattern of methylated anthocyanin variation we observe in different grapes cultivars.

## Conclusions

The aim of this work was to identify and quantify the effect of molecular factors controlling the variation of anthocyanin methylation in grape by combining genetic mapping and functional study. Most previous studies regarding the accumulation of anthocyanin in grape have pointed to the main involvement of the *VvMybA *locus [16,18-21,44]. Our findings confirmed the effect of this locus, but we made an important step forward by revealing the influence of novel factors other than Myb type genes involved in variation of anthocyanin composition. We quantitatively assessed the effect of the *VvAOMT *genes cluster on anthocyanin methylation. Furthermore, we identified new gene variants encoding proteins with slight differences in catalytic properties from other AOMTs described so far, showing allele-guided substrate specificity. This study improves our knowledge on how differences in methylated anthocyanin level may exist independently of total anthocyanin content. The relative independence of these two traits is possible because of the partial functional redundancy between the *VvAOMT *isogenes, among which *VvAOMT*2 "escaped" the control of *VvMybA*. Finally, with a relaxed functional constraint on *VvAOMT*2 while *VvAOMT1 *is still ensuring the persistency of the activity, variation could arise, giving rise to novel phenotypes.

## Methods

### Plant materials

The plant material consisted of three populations: one cross-derived mapping population for QTL mapping and two nested natural populations for the association study. The mapping population (SxG) was a F1 progeny of 191 individuals from a reciprocal cross between clone 73 of Syrah (S) and clone 516 of Grenache (G) replicated in two blocks (A and B) as previously described [20]. Fifty colorful genotypes from the agromorphological core-collection defined by Barnaud et al. [33] were selected to optimally represent the diversity in anthocyanin methylation level. All genomic DNA analyses presented in the study were realized on this collection of 50 colorful cultivars. Within these 50 genotypes, we selected a subset of 32 individuals (listed in Additional file 2) designed through the M-method [37,38] to optimize the variability of the level of anthocyanins methylation. All transcriptomic analyses and sequencing of cDNA performed in the study were realized on this 32 subcore-collection. Mature berries of each genotype were harvested 32 days after véraison.

### Traits analyzed

Grape berries were harvested at the same level of maturity (20° Brix) in 2006 for the SxG progeny and in 2008 for the colorful genotypes and the subcore-collection. The anthocyanin composition of samples was analyzed as described in Fournand et al. [45]. The concentration of delphinidin (Dp), cyanidin (Cy), peonidin (Pn), petunidin (Pt) and malvidin (Mv) derivatives was expressed in mg of malvidin 3-*O-*glucoside equivalent per gram of fresh berry skin. Level of anthocyanin methylation (MT) was calculated as the ratio of methylated/non-methylated anthocyanins. The methylated/non-methylated ratio was log-transformed in order to unskew their distribution. In the SxG sample, the normality of the distribution was checked using the Shapiro-Wilks test for individuals containing anthocyanins (non-white cultivars). The total anthocyanin content was calculated as ln(1 + x) where × is the sum of all anthocyanin compounds. The HPLC data and the MT variable for the subcore-collection are presented in Additional file 2.

### DNA extraction, genotyping and sequencing

Two square centimeters (80-100 mg) discs of fresh young leaves were collected for each genotype. DNA was extracted using a Qiagen DNA Plant Mini Kit (QIAGEN S.A., Courtaboeuf, France) with slight modifications as described in Adam-Blondon et al. (2004). The 191 SxG offsprings have been previously genotyped for 97 SSR markers to cover the genome with minimal inter-marker space (maximum of 10 cM) with the most informative polymorphisms (priority given to 1:1:1:1 segregating markers). An additional marker corresponding to a 200 bp insertion/deletion polymorphism (InDel) in the *VvAOMT*2 locus promoter was genotyped. PCR conditions were 95°C for 7 min, 35 cycles of 95°C (30 s), 58°C (30 s), 72°C (1 min) and a final cycle of 72°C for 5 min. Amplified fragments were run on a 1% agarose gel, stained with ethidium-bromide and photographed under UV light. Amplification primers were designed using the Primer 3 software and are listed in Additional file 10. PCR fragments were amplified, sequenced, and analyzed as described by Le Cunff et al. [46].

### RNA extraction, sequencing and quantitative real-time PCR

For transcriptomic assays, berries were sorted by density on salt gradient between 100 and 120 g/L to ensure homogenous maturity stage within and across all samples and immediately frozen in liquid nitrogen. RNA was extracted from 200 mg of berry powder using RNeasy Plant Mini Kit (QIAGEN S.A., Courtaboeuf, France) following the manufacturer's protocol and then quantified with Ribogreen (Molecular probes, Leiden, The Netherlands). Reverse transcription was performed in triplicate from each sample from 500 ng of purified RNA as described in Fournier-Level et al. [20]. Amplification primers were designed using the Primer 3 software and are listed in Additional file 10. PCR fragments were amplified, sequenced and analyzed as described in Le Cunff et al. [46]. The expression of *VvAOMT *and *VvAOMT*2 was measured for the 32 cultivars showing contrasted methylated anthocyanin berry content. PCR was performed as described in Fernandez et al. [47] by comparing the cycle threshold (CT) of the target gene with that of *VvEF-1 alpha *gene used as an internal standard.

### Framework genetic maps and QTL detection

Framework genetic maps were constructed according to the pseudo-testcross strategy [48] using Carthagene 0.999R software [49] as described in Fournier-Level et al. [20] with an additional marker added corresponding to a 200 bp InDel in *VvAOMT*2 of chromosome 1 segregating in the SxG cross. QTL detection was performed on both parents and consensus maps. Composite interval mapping (CIM) on MT was performed separately on the A and B blocks for the consensus map using MapQTL 4.0 [50], and both blocks together for parental maps using the multi-trait option QTL Cartographer [51]. For CIM on the parental maps, we used the Forward & Backward regression method for cofactor selection with 0.1 as the in-and-out threshold for the *P*-value of the partial F-test. Then, a genome scan was performed with a maximum of five cofactors within a 10 cM window. LOD thresholds corresponding to an experimentwise error rate of 5% were then determined through 1000 permutations. For CIM on the consensus map, we determined the simple interval mapping LOD thresholds through 1000 permutations with a genome-wide error rate of 5%. 99% and 90% confidence intervals were determined through 1000 bootstrap resampling using the R/qtl package.

### Statistical tests

Statistical tests were performed using R software (The R project, USA) following the Shapiro-Wilks method for normality tests and Pearson's for correlation tests. Candidate genes were selected on the grape reference genome sequence according to (1) their presence in the QTL intervals and (2) their homology with genes involved in anthocyanin metabolism. Association tests were carried out using R following the Efficient Mixed Model Association method [34] with kinship matrix estimated using 20 SSR markers scattered along the 19 grape linkage groups [52]. Association tests were performed on each gene in order to determine the highly associated polymorphisms based on *P*-values and adjusted R ^2 ^of a mixed linear model using the kinship matrix as the variance-covariance matrix to control for the genetic background "structure" effect.

### Cloning of *VvAOMT2 *and characterization of recombinant *VvAOMT2*

The full-length cDNA of *VvAOMT*2 was amplified from cDNA of mature berries from two cultivars, Petit Bouschet × Aramon n°4 (PB) and Papadiko (PK), with high-fidelity Taq polymerase (Advantage-HF 2 PCR kit; Clonetech) using the upstream primer 5'-GTGGATCCTCTTCAACCATGTCCAGCTCAAGTCA-3' and the downstream primer 5'-TTGCGGCCGCATAGATTTAGGCTAATAGAGGCGC-3'. The corresponding amplified cDNAs were named PK-AOMT2 (cv. Papadiko) and PB-OMT2 (cv. Petit Bouschet × Aramon n°4). Both amplified cDNAs for *VvAOMT*2 were cloned into the pGEM-T easy vector (Promega) and the resulting plasmids were sequenced to verify that no mutation had been introduced. The recombinant plasmids were then digested by the BamH1/Not1 enzymes, and the resulting restriction fragments were ligated into the pGEX-4 T-2 recombinant vector (GE Healthcare) digested by BamH1/Not1. The AOMT2 proteins were expressed as GST fusion proteins. Recombinant proteins were purified using glutathion Sepharose affinity resin following the pGEX instruction's manual. The characterization of the recombinant proteins was performed after the cleavage of the GST moiety using a site-specific protease whose recognition sequence was located immediately upstream from the multiple cloning sites on the pGEX plasmid and then quantified by SDS-PAGE (Additional file 11). The activity of both PB-AOMT2 and PK-AOMT2 enzymes was then tested *in vitro *using anthocyanins as substrate in the presence of S-adenosyl-L-methionine (SAM) as described in Hugueney et al. [11]. The enzymatic reactions were incubated at 30°C for 1 h 30. For AOMT2 characterization, three independent recombinant protein preparations were tested, and four enzymatic assays were performed with each preparation. Reaction products were analyzed by HPLC-DAD as previously described [45]. Anthocyanins were identified according to their UV-visible absorption spectra and retention time; quantifications were based on peak areas at 520 nm using external calibration with malvidin 3-glucoside.

## Competing interests

The authors declare that they have no competing interests.

## Authors' contributions

AFL and AA wrote the manuscript. AFL, AA, PT and PH designed the study. AFL, AA, CV and PH performed the experiments. PT and PH revised the manuscript. All authors read and approved the final manuscript. All authors declare no conflict of interest.

## Accession numbers

Sequence data from this article have been deposited with the EMBL/GenBank data libraries under accession number # HQ702997 (AOMT2 from Petit Bouschet × Aramon n°4). The sequence Papadiko differs from Petit Bouschet × Aramon n°4 in two SNPs (T127 to C and A388 to T).

## Supplementary Material

Additional file 1 **Summary statistics describing the anthocyanin profiles in the SxG progeny**. The anthocyanin content is expressed in mg of aglycone anthocyanin per g of fresh berry skin. Key: Min, minimum; Qu, quantile; Max, maximum. Click here for file

Additional file 2 **Anthocyanin content and expression profile of VvMybA, VvAOMT and VvAOMT2 in the Core-coll 32 cv**. Anthocyanins content is expressed in mg of anthocyanin per g of fresh skin and expression profile for VvMybA, VvAOMT and VvAOMT2 expressed as--2ΔCt. Click here for file

Additional file 3 **Comparison of the MT variation between the two alleles of the SxG progeny measured in the block B**. The Syrah parent is homozygote for the "strong" allele of VvOMT2 while the Grenache parent is heterozygous with the "weak" and"strong" combination of alleles. Click here for file

Additional file 4 **List of the unigene from the NCBI Vitis EST database present in 1.05 Mbp surrounding the position of maximum LOD for the QTL on the LG1**. Click here for file

Additional file 5 **Comparison of the AOMT1, AOMT2 and AOMT3 amino acid sequences**. Amino acid sequences were aligned using CLUSTAL W. Residues identical to the VvAOMT1 sequence were shaded. Click here for file

Additional file 6 **Specific amplification of AOMT, AOMT2 and AOMT3 on both (A) genomic DNA and (B) coding DNA**. (A) Amplification on genomic DNA (cv. Syrah). (B) Amplification on cDNA of cv. Joubertin (1; 2; 3), cv. Molinara (4; 5; 6) and cv. Petit Bouschet (7; 8; 9) mature berries of AOMT (1; 4; 7), AOMT2 (2; 5; 8) and AOMT3 (3; 6; 9). Click here for file

Additional file 7 **Linkage disequilibrium between polymorphisms in the VvAOMT1 and VvAOMT2 genes for the collection of 50 cv**. R2 are reported on the lower diagonal and P-val from chi-square test for independence are reported on the upper diagonal. Click here for file

Additional file 8 **Quantitative real-time PCR expression profiling of AOMT (black) and AOMT2 (grey) in hairy roots (control and VlmybA1-2 transformed)**. All data are the mean (± SD) of three replicates on two (control) and four (VlmybA1-2 transformed) independent lines. Expression values have been normalized with VvEF1alpha and expressed as relative abundance. Click here for file

Additional file 9 **Polymorphisms in the VvAOMT2 cDNA**. The name of the SNPs corresponds to the nature of the mutation in IUB code followed by the position on the coding sequence. Non-synonymous mutations are presented in bold with a red arrow, mutations in the 3'UTR non-coding sequence are presented with dashed arrows and not reported at the right scale. Click here for file

Additional file 10 **PCR primers used for real-time PCR and expected size for amplified fragments**. Key: gDNA (genomic DNA); cDNA (coding DNA). Click here for file

Additional file 11 **SDS-PAGE of the recombinant AOMTs obtained from cv**. Papadiko (PK-AOMT) and cv. Petit Bouschet × Aramon n°4 (PB-AOMT). Lanes: (1) protein size markers; (2) purified PK-AOMT after GST cleavage. (3) purified PB-AOMT after GST cleavage. The molecular weights of the markers are indicated in kDa. Click here for file
