# Supplementary figures and images for: Genetic mechanisms underlying the methylation level of anthocyanins in grape (Vitis vinifera L.)
Source: BMC Plant Biol. 2011 Dec 15;11:179. doi: 10.1186/1471-2229-11-179 (PMC3264682; doi:10.1186/1471-2229-11-179)

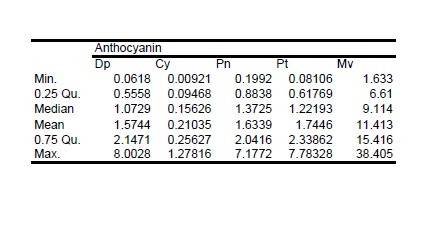

Supplement: Additional file 1 — Summary statistics describing the anthocyanin profiles in the SxG progeny. The anthocyanin content is expressed in mg of aglycone anthocyanin per g of fresh berry skin. Key: Min, minimum; Qu, quantile; Max, maximum. [file 1471-2229-11-179-S1.BMP]

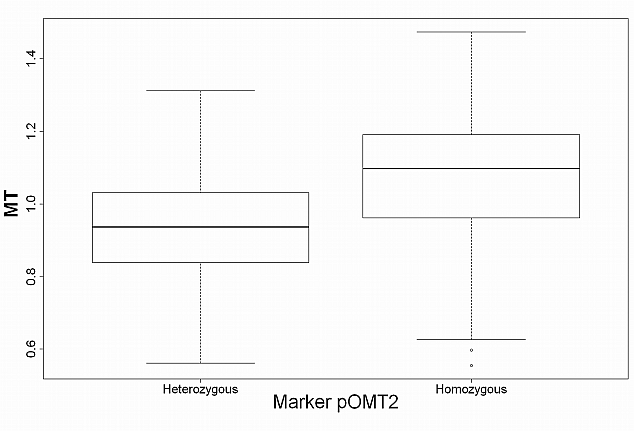

Supplement: Additional file 3 — Comparison of the MT variation between the two alleles of the SxG progeny measured in the block B. The Syrah parent is homozygote for the "strong" allele of VvOMT2 while the Grenache parent is heterozygous with the "weak" and"strong" combination of alleles. [file 1471-2229-11-179-S3.BMP]

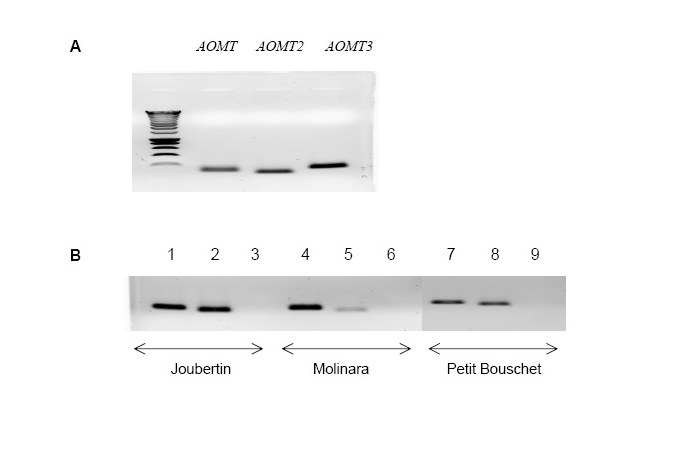

Supplement: Additional file 6 — Specific amplification of AOMT, AOMT2 and AOMT3 on both (A) genomic DNA and (B) coding DNA. (A) Amplification on genomic DNA (cv. Syrah). (B) Amplification on cDNA of cv. Joubertin (1; 2; 3), cv. Molinara (4; 5; 6) and cv. Petit Bouschet (7; 8; 9) mature berries of AOMT (1; 4; 7), AOMT2 (2; 5; 8) and AOMT3 (3; 6; 9). [file 1471-2229-11-179-S6.BMP]

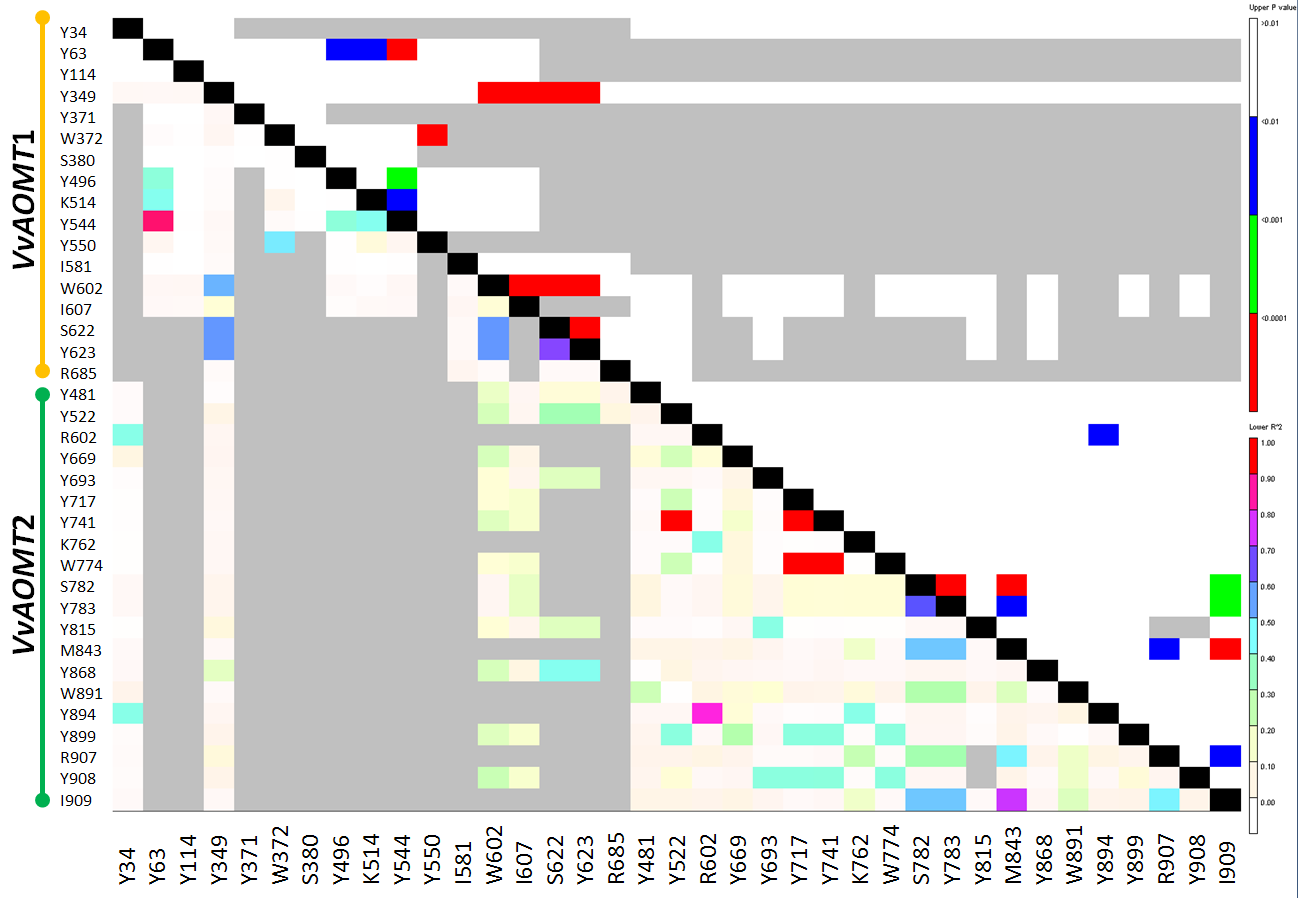

Supplement: Additional file 7 — Linkage disequilibrium between polymorphisms in the VvAOMT1 and VvAOMT2 genes for the collection of 50 cv. R2 are reported on the lower diagonal and P-val from chi-square test for independence are reported on the upper diagonal. [file 1471-2229-11-179-S7.BMP]

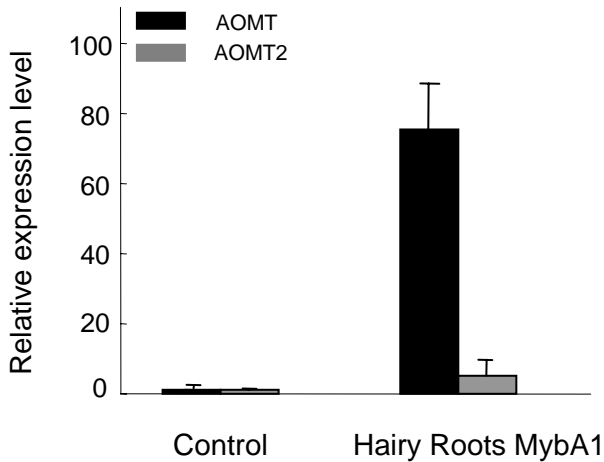

Supplement: Additional file 8 — Quantitative real-time PCR expression profiling of AOMT (black) and AOMT2 (grey) in hairy roots (control and VlmybA1-2 transformed). All data are the mean (± SD) of three replicates on two (control) and four (VlmybA1-2 transformed) independent lines. Expression values have been normalized with VvEF1alpha and expressed as relative abundance. [file 1471-2229-11-179-S8.PDF]

VvAOMT2 (GSVIVT01010468001)

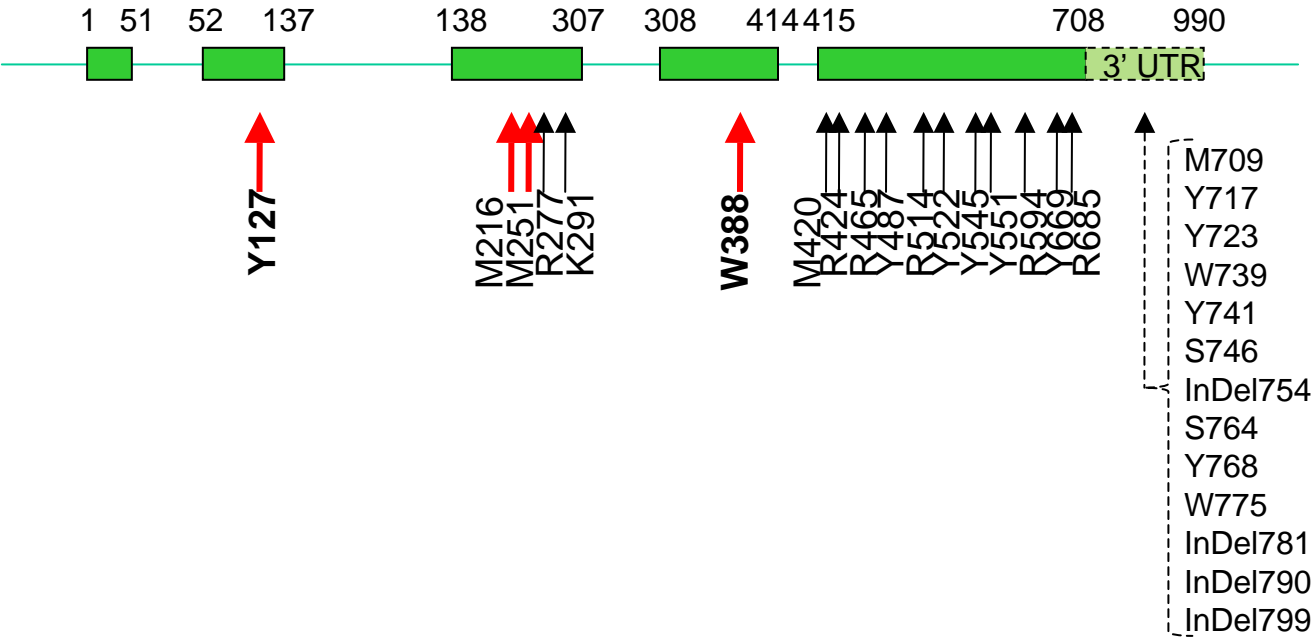

Supplement: Additional file 9 — Polymorphisms in the VvAOMT2 cDNA. The name of the SNPs corresponds to the nature of the mutation in IUB code followed by the position on the coding sequence. Non-synonymous mutations are presented in bold with a red arrow, mutations in the 3'UTR non-coding sequence are presented with dashed arrows and not reported at the right scale. [file 1471-2229-11-179-S9.PDF]

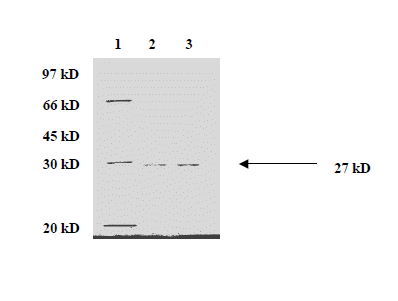

Supplement: Additional file 11 — SDS-PAGE of the recombinant AOMTs obtained from cv. Papadiko (PK-AOMT) and cv. Petit Bouschet × Aramon n°4 (PB-AOMT). Lanes: (1) protein size markers; (2) purified PK-AOMT after GST cleavage. (3) purified PB-AOMT after GST cleavage. The molecular weights of the markers are indicated in kDa. [file 1471-2229-11-179-S11.BMP]
