# Supplementary material for: Genetic mechanisms underlying the methylation level of anthocyanins in grape (Vitis vinifera L.)
Source: BMC Plant Biol. 2011 Dec 15;11:179. doi: 10.1186/1471-2229-11-179 (PMC3264682; doi:10.1186/1471-2229-11-179)
Supplement: Additional file 2 — Anthocyanin content and expression profile of VvMybA, VvAOMT and VvAOMT2 in the Core-coll 32 cv. Anthocyanins content is expressed in mg of anthocyanin per g of fresh skin and expression profile for VvMybA, VvAOMT and VvAOMT2 expressed as--2ΔCt. [file 1471-2229-11-179-S2.PDF]

Additional File 2 Anthocyanin content and expression profile of *VvMybA*, *VvAOMT* and *VvAOMT*2 in the Core-coll 32 cv. Anthocyanins content is expressed in mg of anthocyanin per g of fresh skin and expression profile for *VvMybA*, *VvAOMT* and *VvAOMT*2 expressed as -2ΔCt.

Name	*Dp-3-gl*	*Cy-3-gl*	*Pt-3-gl*	*Pn-3-gl*	*Mv-3-gl*	*Dp-3-gl-ac*	*Cy-3-gl-ac*	*Pt-3-gl-ac*	*Pn-3-gl-ac*	*Dp-3-gl-pcoum*	*Mv-3-gl-ac*	*Cy-3-gl-pcoum*	*Pt-3-gl-pcoum*	*Pn-3-gl-pcoum*	*Mv-3-gl-pcoum*	-2ΔCt e*MybA*	-2ΔCt e*AOMT*	-2ΔCt e*AOMT2*
Alabar Noir	0.131	0.015	0.171	0.548	4.000	0.004	0.011	0.011	0.013	0.047	0.199	0.006	0.025	0.142	1.266	-5.558	-1.169	-10.534
Chatus Noir	1.827	0.531	1.479	1.584	4.938	0.020	0.002	0.032	0.029	0.208	0.138	0.042	0.077	0.155	0.462	-3.479	-0.064	-1.312
Dalmasso XVIII = Viola	0.578	1.415	0.397	2.246	0.878	0.004	0.001	0.001	0.012	0.015	0.012	0.029	0.006	0.060	0.028	-3.585	-2.545	-10.194
Estaca Saoumo	0.743	0.157	0.926	0.762	6.192	0.008	0.010	0.047	0.016	0.306	0.281	0.052	0.348	0.358	3.306	-6.223	-2.018	-9.973
Faux Courbu	2.167	0.559	1.319	0.768	3.814	0.080	0.016	0.053	0.022	0.125	0.229	0.023	0.036	0.039	0.202	-5.918	-1.385	-2.547
Flame Seedless	0.141	0.946	0.037	0.398	0.047	0.001	0.001	0.002	0.002	0.001	0.002	0.027	0.002	0.018	0.001	2.262	-3.165	-8.340
Giro	0.588	0.126	0.537	0.479	2.314	0.015	0.006	0.029	0.022	0.418	0.109	0.169	0.350	0.340	1.588	-12.707	-2.860	-6.450
Gros Colaman	0.292	0.016	0.370	0.354	3.969	0.009	0.001	0.013	0.004	0.091	0.126	0.006	0.063	0.069	0.918	-5.175	-1.415	-4.062
Gueuche Noir	0.115	0.053	0.174	0.775	3.156	0.001	0.001	0.025	0.001	0.042	0.060	0.039	0.045	0.174	1.013	-6.152	-0.465	-3.417
Joubertin	3.814	0.911	2.624	2.056	8.073	0.367	0.090	0.426	0.209	0.503	1.008	0.195	0.267	0.312	1.374	-7.588	-0.934	-3.159
Kincsem	0.024	4.549	0.007	0.089	0.006	0.001	0.001	0.001	0.001	0.001	0.001	0.001	0.001	0.001	0.001	-4.990	-5.182	-8.787
Lledoner pelut	0.139	0.100	0.160	0.728	1.856	0.001	0.003	0.007	0.012	0.017	0.047	0.013	0.016	0.111	0.323	-8.378	-4.568	-12.379
Meunier	0.189	0.066	0.297	1.206	3.771	0.001	0.014	0.025	0.001	0.001	0.001	0.001	0.001	0.001	0.001	-5.720	-1.178	-8.687
Molinara Gorda	0.095	1.009	0.033	0.634	0.095	0.001	0.006	0.002	0.007	0.001	0.002	0.049	0.002	0.030	0.004	-5.034	-3.384	-11.096
Molinera	6.879	3.366	3.441	4.113	8.627	0.988	0.362	0.664	0.490	0.509	1.615	0.307	0.270	0.441	1.124	-6.024	-1.929	-1.882
Morenillo	1.153	0.344	0.899	1.307	4.760	0.016	0.004	0.034	0.030	0.108	0.236	0.073	0.103	0.309	1.037	-3.137	1.623	-1.155
Mourisco Tinto	0.304	0.034	0.323	0.216	2.306	0.007	0.001	0.015	0.003	0.143	0.083	0.027	0.142	0.112	1.406	-0.951	1.003	-6.450
Muscat Rouge de Madeire	0.031	0.078	0.030	0.532	0.484	0.001	0.002	0.002	0.003	0.003	0.002	0.011	0.001	0.046	0.032	-6.167	-3.903	-10.108
Negrara Trentina	0.696	2.190	0.673	2.913	1.714	0.012	0.006	0.016	0.021	0.031	0.007	0.135	0.013	0.125	0.057	-0.444	-2.666	-3.161
Nerello Mascallese	1.158	1.833	0.752	1.216	1.343	0.001	0.001	0.001	0.001	0.001	0.001	0.018	0.001	0.019	0.019	0.438	-3.677	-10.348
Ouliven	0.031	0.039	0.036	0.196	0.562	0.001	0.001	0.001	0.007	0.013	0.025	0.012	0.009	0.083	0.260	-4.415	-2.295	-10.527
Papadiko	0.683	0.285	0.720	0.940	2.858	0.060	0.019	0.104	0.087	0.305	0.463	0.147	0.283	0.400	1.480	2.517	-1.693	-9.160
Petit Bouschet	0.147	0.071	0.174	1.944	3.190	0.003	0.002	0.015	0.022	0.014	0.038	0.042	0.021	0.400	0.660	-2.256	0.201	-7.060
Petit Bouschet x Aramon n°1	0.127	0.072	0.163	1.976	3.181	0.003	0.003	0.012	0.022	0.022	0.058	0.022	0.021	0.420	0.639	-5.945	-0.267	-8.478
Petit Bouschet x Aramon n°4	0.399	0.523	0.407	7.158	4.288	0.006	0.011	0.029	0.102	0.064	0.138	0.091	0.050	1.231	1.020	-1.406	2.099	-7.210
Ribol	0.894	2.483	0.489	2.653	0.784	0.005	0.014	0.001	0.032	0.038	0.007	0.249	0.018	0.259	0.067	-6.330	-3.475	-5.677
Roussaitis	0.006	0.007	0.007	0.045	0.074	0.002	0.002	0.002	0.002	0.002	0.001	0.005	0.003	0.022	0.028	-11.573	-2.920	-7.391
Saint Laurent = Negret n°1	0.082	0.034	0.104	0.470	1.716	0.001	0.001	0.008	0.011	0.043	0.083	0.014	0.050	0.220	1.155	-6.148	-1.322	-4.192
Salba	2.740	0.815	2.460	3.427	9.387	0.347	0.122	0.432	0.604	0.668	1.804	0.224	0.428	0.899	2.359	-6.242	-1.775	-10.879
Travisana Nera	0.704	0.212	1.066	0.301	4.877	0.081	0.044	0.228	0.067	0.440	1.135	0.212	0.519	0.169	2.457	-5.762	-0.961	-9.843
Trollingi Feher	0.017	0.029	0.014	0.040	0.069	0.002	0.003	0.004	0.011	0.023	0.009	0.020	0.006	0.042	0.054	-8.565	-3.978	-5.473
Verdejo	0.937	0.250	0.869	1.140	4.903	0.104	0.027	0.158	0.157	0.247	0.918	0.151	0.260	0.425	1.701	-7.854	-3.249	-5.865
