# Supplementary material for: Genetic mechanisms underlying the methylation level of anthocyanins in grape (Vitis vinifera L.)
Source: BMC Plant Biol. 2011 Dec 15;11:179. doi: 10.1186/1471-2229-11-179 (PMC3264682; doi:10.1186/1471-2229-11-179)
Supplement: Additional file 4 — List of the unigene from the NCBI Vitis EST database present in 1.05 Mbp surrounding the position of maximum LOD for the QTL on the LG1. [file 1471-2229-11-179-S4.PDF]

Locus Name (12X grape genome)	Chromosome	Start (bp)	End (bp)	Candidate genes
GSVIVT01010360001	chr1	19784551	19799430	
GSVIVT01010361001	chr1	19802033	19816465	
GSVIVT01010362001	chr1	19816505	19833346	
GSVIVT01010363001	chr1	19834919	19840992	
GSVIVT01010364001	chr1	19850530	19856793	
GSVIVT01010365002	chr1	19857100	19861937	
GSVIVT01010368001	chr1	19877398	19881935	
GSVIVT01010369001	chr1	19882932	19887719	
GSVIVT01010370001	chr1	19888622	19890374	
GSVIVT01010371001	chr1	19893130	19896199	
GSVIVT01010372001	chr1	19913049	19914939	
GSVIVT01010373001	chr1	19921837	19923437	
GSVIVT01010374001	chr1	19925332	19930565	
GSVIVT01010375001	chr1	19942746	19949762	
GSVIVT01010376001	chr1	19953545	19957705	
GSVIVT01010378001	chr1	19997292	20000950	
GSVIVT01010379001	chr1	20003391	20003813	
GSVIVT01010380001	chr1	20007761	20013929	
GSVIVT01010382001	chr1	20019343	20022287	
GSVIVT01010390001	chr1	20078603	20081514	
GSVIVT01010391001	chr1	20085777	20097440	
GSVIVT01010392001	chr1	20100521	20102317	
GSVIVT01010394001	chr1	20110753	20122015	
GSVIVT01010395001	chr1	20143753	20144505	
GSVIVT01010397001	chr1	20152663	20154159	
GSVIVT01010404001	chr1	20229630	20231128	
GSVIVT01010416001	chr1	20363959	20365455	
GSVIVT01010429001	chr1	20529405	20530313	
GSVIVT01010431001	chr1	20542070	20544780	
GSVIVT01010436001	chr1	20564870	20568070	
GSVIVT01010437001	chr1	20569149	20570248	
GSVIVT01010439001	chr1	20592975	20594160	
GSVIVT01010441001	chr1	20625401	20652989	
GSVIVT01010442001	chr1	20653444	20660585	
GSVIVT01010443001	chr1	20661352	20663794	
GSVIVT01010444001	chr1	20663797	20665821	
GSVIVT01010452001	chr1	20714759	20734567	
GSVIVT01010454001	chr1	20746045	20750153	
GSVIVT01010461001	chr1	20779156	20780679	
GSVIVT01010463001	chr1	20788641	20805175	
GSVIVT01010464001	chr1	20813288	20814946	
GSVIVT01010466001	chr1	20818548	20820093	*VvAOMT3*
GSVIVT01010467001	chr1	20822810	20827657	*VvAOMT2*
GSVIVT01010468001	chr1	20828171	20830796	*VvAOMT1*
GSVIVT01010469001	chr1	20835862	20852573	
GSVIVT01010470001	chr1	20854747	20855013	
