# Supplementary material for: Genetic mechanisms underlying the methylation level of anthocyanins in grape (Vitis vinifera L.)
Source: BMC Plant Biol. 2011 Dec 15;11:179. doi: 10.1186/1471-2229-11-179 (PMC3264682; doi:10.1186/1471-2229-11-179)
Supplement: Additional file 5 — Comparison of the AOMT1, AOMT2 and AOMT3 amino acid sequences. Amino acid sequences were aligned using CLUSTAL W. Residues identical to the VvAOMT1 sequence were shaded. [file 1471-2229-11-179-S5.PDF]

```
AOMT1   MSSSSHRGILKTEALTKYLLETSAYPREHEQLKGLREATVEKHHKYWSLMN 50
AOMT2   MSSSSHKGILKTEALTKYILETSAYPREHEQLKGLREATVEKHHKYWSLMN 50
AOMT3   MSSLIHKGILKTEALTKYILETSAYPREHEQLRGLREATVEKQEFWSIMN 50
        ***  *:***********:*************:*********:::**:**

AOMT1   VPVDEGLFISMLLKIMNAKKTIELGVFTGYSLLATALALPQDGKIIAVDP 100
AOMT2   VPVDEGQFISMLLKIMNAKKTIELGVFTGYSLLATALALPQDGKIIAVDP 100
AOMT3   VPVDEGLFISMLLKLMNVKKTIELGVFTGYSLLATPLALPQDGKIIAVDP 100
        ****** *******:**.*******************.************

AOMT1   DKEAYQTGVPFIKKAGVEHKINFIQSDAMSVLNDLIADGKEEGTLDFAMV 150
AOMT2   DKEAYQTGVPFIKKAGVEHKINFIQSDAMTVLNDLIADGNEEGTLDFAFV 150
AOMT3   DKEAYQIGVPFIKKPGVEHKINFIQSDAMSVLNDLIADGNEEGTLDFAFV 150
        ****** *******.**************:*********:********:*

AOMT1   DADKENYLNYHELLLKLVRVGGIIAYDNTLWFGSVARSEEEEMMDFERAG 200
AOMT2   DADKENYLNYHELLLKLVRVGGIIAYDNTLWFGSVARSEEEEMMDFERAG 200
AOMT3   DADKENYLNYHELLLKLVRVGGIIAYDNTLWFGSVARSEEEEMMDFERAG 200
        **************************************************

AOMT1   RVHLMKLNKFLASDPRVELSHLSIGDGVALCRRLY- 235
AOMT2   RVHLMKLNKFLASDPRVELSHLSIGDGVALCRRLY- 235
AOMT3   RVHLMKLNKFLASDPRVELSHLSIGDGIALCRRLY- 235
        ***************************:*******
```
