# Supplementary material for: Genetic mechanisms underlying the methylation level of anthocyanins in grape (Vitis vinifera L.)
Source: BMC Plant Biol. 2011 Dec 15;11:179. doi: 10.1186/1471-2229-11-179 (PMC3264682; doi:10.1186/1471-2229-11-179)
Supplement: Additional file 10 — PCR primers used for real-time PCR and expected size for amplified fragments. Key: gDNA (genomic DNA); cDNA (coding DNA). [file 1471-2229-11-179-S10.DOC]

**Amplicon name** **Matrix type** **Sequence of the forward** **Fragment**

**and reverse primers** **length**

Promo VvAOMT2 gDNA F 5’GTGGTGGTGGAGGTAGCAAT 1178 bp

R 5'-AAGCCTGCCGTACAAGAAAA

VvAOMT1 gDNA F 5'-CCTGTCGATGAGGGACTGTT 908 bp

R 5'-AAATACAAATACAAATACAA

VvAOMT2 gDNA F 5'-CCTGTCGATGAGGGACAGTT 1094 bp

R 5'-AGGGAGAATGAATGAAAC

VvAOMT2 cDNA F 5'-ATGTCCAGCTCAAGTCA 990 bp

R 5'-AGGGAGAATGAATGAAAC

AOMT cDNA F 5’ CTCTGCAGGCGCCTCTATTA 138 bp

R 5’ CCCAAAACAGAGTCTGGACA

AOMT2 cDNA F 5’ CTCTGCAGGCGCCTCTATTA 109 bp

R 5’ GTTCAAATACAAATGGAAATCCGTCAA
